# Disparity of the Treatment of Unresectable Non-small Cell Lung Cancer Regarding Chemotherapy: A Systematic Review and Meta-Analysis

**DOI:** 10.7759/cureus.60635

**Published:** 2024-05-19

**Authors:** Ryuichi Ohta, Chiaki Sano

**Affiliations:** 1 Community Care, Unnan City Hospital, Unnan, JPN; 2 Community Medicine Management, Shimane University Faculty of Medicine, Izumo, JPN

**Keywords:** treatment outcome, urban population, rural population, healthcare disparities, chemotherapy, non-small cell lung carcinoma

## Abstract

This study investigates disparities in chemotherapy treatment for unresectable non-small cell lung cancer (NSCLC) between urban and rural populations. Despite advancements in NSCLC treatments enhancing survival, significant inequities persist, notably in rural areas where access to care is often limited, resulting in poorer outcomes. Through a systematic review and meta-analysis, we analyzed data from selected studies that compare chemotherapy access and usage between these populations from 2010 to 2024. Our findings indicate that rural patients are consistently less likely to receive advanced chemotherapy treatments than urban counterparts, with a pooled odds ratio of 0.91 (95% confidence interval (CI): 0.83-1.00), suggesting a marginal but noticeable disparity. This highlights a crucial gap in healthcare provision, underscoring the need for policy interventions and improved healthcare practices to ensure equitable treatment access. This research calls for further investigation into socioeconomic and cultural factors contributing to these disparities to inform targeted improvement strategies.

## Introduction and background

Lung cancer continues to be a leading cause of cancer-related deaths worldwide, with an estimated 1.8 million fatalities annually, according to the World Health Organization [1]. Among the various types, non-small cell lung cancer (NSCLC) is particularly prevalent, accounting for approximately 84% of all lung cancer cases [1,2]. The incidence of NSCLC is notably increasing in middle-aged and older populations, extending even to those without traditional risk factors, such as smoking [3]. This trend is alarming, as advanced stages of NSCLC, which are often the case at diagnosis, complicate treatment options [3]. The evolution of chemotherapy over the past decades has significantly enhanced the quality of life and survival rates for patients with advanced NSCLC, underscoring the critical role of accessible treatment modalities [4].

Despite the therapeutic advancements, significant disparities in the delivery and efficacy of chemotherapy for patients with unresectable NSCLC are evident, particularly across different geographic regions [5]. Epidemiological studies highlight that while the overall five-year survival rate for lung cancer has improved to around 19%, rural populations continue to experience suboptimal outcomes compared to urban areas [6,7]. The National Cancer Institute reports that rural cancer patients have a 19% higher mortality rate than urban patients, indicating a severe access and quality gap in oncological care [8]. This disparity impacts survival rates and quality of care, with rural patients often receiving less frequent and less aggressive treatment protocols [9]. The factors contributing to these disparities are multifaceted, involving socioeconomic, logistical, and systemic healthcare inefficiencies that disproportionately affect rural residents.

This research aims to explore the following question: What are the specific disparities in treating unresectable NSCLC concerning chemotherapy between urban and rural populations? This study aims to comprehensively analyze and synthesize data from existing studies examining the treatment disparities of unresectable NSCLC concerning chemotherapy. By focusing on both national and international epidemiological data, such as treatment access rates and survival outcomes from various regions, this study intends to document and quantify these disparities comprehensively. For example, research indicates that rural patients in some regions are up to 30% less likely to receive advanced chemotherapeutic agents than their urban counterparts, and their access to newer, targeted therapies could be much better [10]. This analysis aims to provide robust evidence that can inform healthcare policy and practice, aiming to reduce these disparities and improve equitable access to effective NSCLC treatments for all patients, regardless of geographic location. Through this scholarly work, we seek to contribute significantly to the ongoing efforts to enhance cancer care and ensure that advancements in treatment are accessible to every segment of the population.

## Review

Methods

This study followed guidelines stipulated in the Preferred Reporting Items for Systematic Reviews and Meta-analyses (PRISMA) statements [11]. We searched four databases (PubMed, the Cochrane Library, Google Scholar, and Web of Science) for original articles regarding the difference in medical treatments between urban and rural areas in unresectable NSCLC from April 2010 to August 2024. The study duration was decided by referencing the history of the advancement of chemotherapy [12]. Regarding articles written in English, our search strategy was based on the following title/abstract keywords: (((lung) AND (carcinoma)) AND (rural)) AND (urban). We searched Japanese articles based on the following title/abstract keywords. The reference lists of relevant studies were also reviewed to identify research that might have been missed in the database search.

Study selection

The inclusion and exclusion criteria are listed in Table 1.

**Table 1:** Inclusion and exclusion criteria. NSCLC, non-small cell lung cancer; SCLC, small cell lung cancer

Criteria	Inclusion	Exclusion
Population	Unresectable NSCLC (Stage 3B and 4)	SCLC and NSCLC (Stage 1 to 3A)
Intervention	Chemotherapy	Other interventions except for chemotherapy
Type of study	Cross-sectional and cohort study	Other study design, non-empirical studies (editorials, news)
Other	Abstract available, year of publication >2010, full text available in English	Abstract not available, full text not available in English

We included original articles. We also excluded conference presentations and original articles. The duplicated articles in the search result were also excluded.

Data extraction

The first authors independently conducted literature searches and data extraction, and any discrepancies were resolved through discussion. In this study, databases were searched for original studies regarding the difference in medical treatments between urban and rural areas in unresectable NSCLC. Studies conducted without clearly describing the aim, participants, or outcomes were excluded.

The first investigator (RO) extracted information regarding the difference in medical treatments between urban and rural areas in unresectable NSCLC. The concrete extracted contents were publication year, country, first author name, title, purpose, study design, participants, the number of rural and urban patients, and the number of chemotherapy participants. The second investigator (CS) checked the extracted information. The extracted information was synthesized through meta-ethnography.

Statistical analysis

The quality of each study was assessed based on the Best Evidence Medical Education (BEME) scale (1 to 5): Grade 1 indicates that no definite conclusions could be drawn, that is, the data are not significant; Grade 2 indicates that the results are ambiguous, but there appears to be a trend; Grade 3 indicates that conclusions could probably be drawn based on the results; Grade 4 indicates that the results are precise and very likely to be accurate; Grade 5 indicates that the results are unequivocal [13]. The included studies were subjected to a thorough descriptive analysis. This involved summarizing each study in terms of its essential characteristics and findings. The data were compiled into a comprehensive table, providing an overview of the scope and nature of existing research on the topic. 

We performed a meta-analysis using the random-effects model to account for potential heterogeneity among studies, which was quantified using the I² statistic. I² value of 0% indicates no observed heterogeneity, while values greater than 50% suggest substantial heterogeneity. In cases of significant heterogeneity, sources were further explored via subgroup analyses. Data from individual studies were pooled to compute the overall effect sizes, represented as odds ratios (ORs) with 95% confidence intervals (CIs) for dichotomous outcomes and mean differences (MDs) or standardized mean differences (SMDs) for continuous outcomes. The Mantel-Haenszel method was employed for dichotomous data and the inverse variance method for continuous data. Publication bias was assessed visually using funnel plots and quantitatively through Egger's regression test. The trim and fill method was utilized to adjust the effect estimates if publication bias was detected. All statistical analyses were performed using EZR (Saitama Medical Center, Jichi Medical University, Saitama, Japan), which is a graphical user interface for R (The R Foundation, Vienna, Austria) [14]. A p-value less than 0.05 was considered statistically significant, except where expressly noted.

Results

Selection of the Included Articles

**Figure 1:**
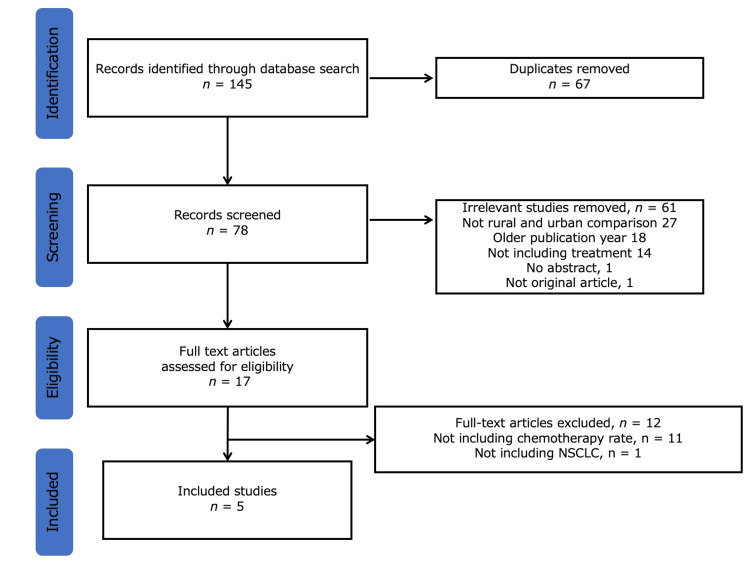
Selection flow of the included articles. NSCLC, non-small cell lung cancer

Of the 145 studies analyzed, 67 were excluded because of duplication. After reviewing the abstracts, 61 studies were excluded for the following reasons (no rural and urban comparison, 27; older publication year, 18; not including treatment, 14; no abstract, 1; not an original article, 1). Seventeen articles were assessed for eligibility, and five were included in the final analysis after excluding 13 articles based on the inclusion and exclusion criteria (Figure 1).

Demographics of the Included Articles

The included articles are shown in Table 2.

**Table 2:** Demographics of the included articles NSCLC, non-small cell lung cancer

Article	Purpose	Country	Design	Results
Steele CB et al., 2011 [15]	To examine urban/rural patterns in receipt of treatment for NSCLC among black and white Medicare beneficiaries in Alabama	United States of America	Retrospective cohort study	Urban and rural white beneficiaries underwent surgical resection more frequently than their black counterparts. Despite controlling for demographic and health variables, significant racial disparities persisted, with Black patients having notably lower odds of receiving surgery compared to white patients in both urban and rural settings.
Johnson AM et al., 2014 [16]	To measure the extent to which geographic residency status and the social environment are associated with disease stage at diagnosis, receipt of treatment, and five-year survival for patients diagnosed with NSCLC	United States of America	Retrospective cohort study	Rural and suburban residents had higher chances of having unstaged disease than urban dwellers. Rural residents were less likely to receive radiotherapy and chemotherapy. Lower educational levels and higher deprivation in communities correlated with reduced treatment access and poorer survival rates. However, rural residents with early-stage disease had a lower mortality risk when treatments were considered.
Thomas AA et al., 2017 [17]	To assess the relationship between relative survival and patients' area of residence, considering surgery receipt and area socioeconomic level	Ireland	Retrospective cohort study	Urban residents were more likely to receive surgery compared to rural residents. However, rural residence was associated with a lower excess mortality for all cases and non-surgical cases specifically, indicating a survival advantage for rural patients despite lower surgical rates.
Ray MA et al., 2020 [18]	To clarify rurality and interact with NSCLC care and outcome disparities	United States of America	Retrospective cohort study	Of the 6,259 patients, 47% reside in rural areas. Those attending urban institutions—urban or rural residents—were more likely to receive stage-preferred treatment and had lower mortality risks than rural residents at rural facilities. Urban institutions offered a survival advantage across all stages of treatment, with even late-stage patients benefiting significantly. Adjustments were made for insurance, age, and clinical stage in the analysis.
Logan CD et al., 2022 [19]	To clarify differences in survival for surgically treated rural and small-town patients compared to those from urban and metropolitan areas	United States of America	Retrospective cohort study	In a study of 366,373 surgically treated NSCLC patients, 12.4% were from rural/small-town areas linked to lower income and education. These patients had to travel further for treatment and had lower survival rates at one, five, and 10 years compared to their urban counterparts. Increased travel distance and living in rural/small-town areas were associated with a higher risk of death, underscoring significant disparities in outcomes based on geographic location and accessibility.

Regarding study design, all studies used retrospective cohort studies. Regarding study settings, four studies were from the United States of America (USA), and one was from Ireland. All the studies contained the disparity of chemotherapy treatment between rural and urban contexts.

Results of the Meta-Analysis

**Figure 2:**
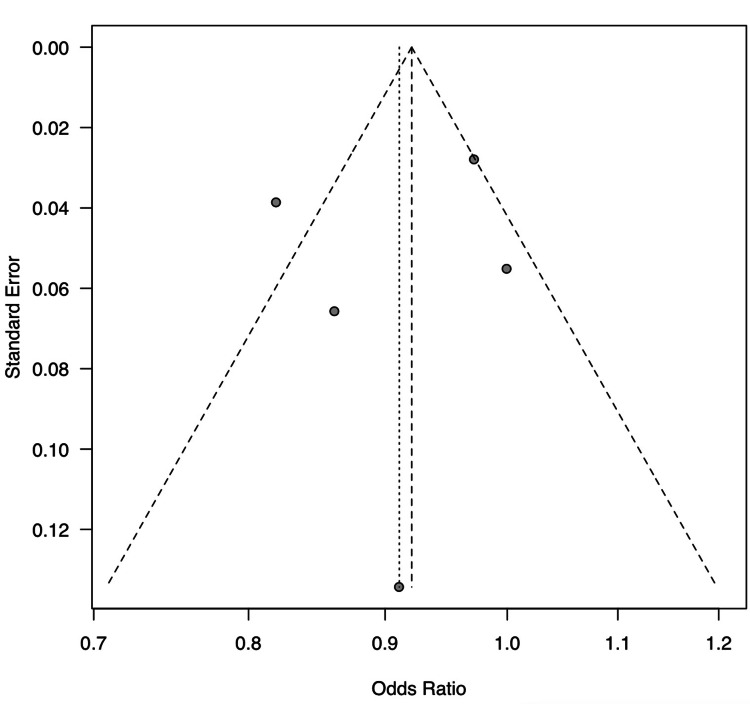
Funnel plot for the confirmation of the heterogeneity

In a meta-analysis aimed at exploring the disparities in chemotherapy treatment for unresectable NSCLC between rural and urban settings, the forest plot indicates a combined total of 15,950 events (chemotherapy usage) in the experimental (rural) group and 74,463 in the control (urban) group. The individual study weights in the random-effects model varied from 8.5% to 27.0%, reflecting the variance in the size and impact of each study. The heterogeneity was significant (I² = 75%, tau² = 0.0072, p < 0.01), as described in the funnel plot analysis (Figure 2).

**Figure 3:**
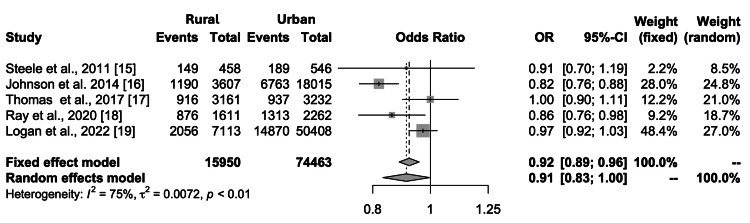
Result of the meta-analysis regarding the disparity of the treatment of chemotherapy between rural and urban contexts. OR, odds ratio

The results warranted using the random-effects model, which showed a pooled odds ratio of 0.91 (95% CI: 0.83-1.00). This indicates a trend where patients with unresectable NSCLC in rural areas may receive chemotherapy less frequently than those in urban areas (Figure 3).

Discussion

Our study underscores significant disparities in the delivery of chemotherapy for unresectable NSCLC between rural and urban patients, reflecting an inequitable healthcare landscape where geographic location plays a pivotal role in the accessibility and quality of cancer care. The pooled OR of 0.91 (95% CI: 0.83-1.00) from our meta-analysis indicates that rural patients are less likely to receive chemotherapy compared to their urban counterparts, consistent with earlier findings highlighting the systematic disadvantages rural residents face in accessing life-saving treatments.

The research conducted by Ray et al. (2020) provides insight into these disparities, noting that rural cancer patients who receive treatment in urban institutions experience better outcomes than those treated in rural settings [18]. This difference can be attributed to the superior quality and broader range of treatment options available in urban centers, which are crucial for improving patient prognosis [20]. In medical care, accessibility is vital for effectively treating diseases [21]. As this systematic review shows, rural areas may lack access to chemotherapy. Fewer patients may not access effective chemotherapy, which worsens their mortality [22].

Furthermore, Logan et al. (2022) emphasized the additional burden of travel for rural residents, who often have to traverse greater distances for care. Thus, they face increased challenges in maintaining continuity of treatment and ultimately experience lower survival rates [19]. In advanced medical situations, chemotherapy needs continuity of care in advanced medical institutions [23]. To improve rural oncology care, including NSCLC, rural hospitals should include advanced cancer care and collaborate with advanced medical institutions.

The socioeconomic aspect of these disparities cannot be overlooked. Johnson et al. (2014) revealed a direct correlation between educational attainment and survival outcomes in rural areas, suggesting that lower educational levels, often linked with reduced economic opportunities, adversely affect health outcomes [16]. Rural people’s help-seeking behaviors (HSB) may also cause ineffective usage of medical care, leading to the delay and less usage of chemotherapy among NSCLC patients [24]. These interconnections imply that any effective intervention must tackle the medical disparities and the underlying socioeconomic factors contributing to the uneven landscape of cancer care [25]. In particular, older patients in rural contexts may lack effective HSBs in previous articles. Culturally, rural patients tend to endure their symptoms, delaying the detection of cancers [24,26]. Respecting their aspects, educational intervention regarding HSBs should be promoted to improve the disparities.

The notable heterogeneity observed in our meta-analysis (I² = 75%) indicates a significant variance in the degree of disparity across different studies, which reflects the complex interaction of multiple factors, such as healthcare policies, local infrastructure, and the availability of specialized care. Rural healthcare can be affected by the country’s administration and cultural and racial disparities [27,28]. In this study, most studies are from the USA, which has different independent states with different medical administration and policies. In addition, there are differences in rurality dependent on the geographical conditions [29]. These elements greatly influence the implementation and efficacy of cancer treatments and point to the need for nuanced, context-specific approaches to healthcare provision.

This study has several limitations. As a limitation, four out of five studies analyzed for this meta-analysis are from the USA and represent a specific kind of healthcare delivery system. The findings primarily pertain to the settings and populations in the meta-analysis, which may limit their external validity to other regions or healthcare systems with different infrastructural or socioeconomic contexts. The substantial heterogeneity among the included studies suggests variations in study design, population demographics, and local healthcare practices, which could affect the consistency and reliability of the pooled outcomes. The reliance on retrospective cohort studies and the quality of data reported in these studies could introduce biases, particularly in how rural and urban classifications are made and the completeness of chemotherapy treatment records. Our study primarily focuses on logistical and infrastructural aspects of healthcare disparities and less on the socioeconomic and cultural barriers that may also significantly impact access to care [30]. These factors include health literacy, cultural beliefs about medicine, and stigma associated with cancer treatment. This study did not directly assess the impact of local, state, and federal health policies, which could play a critical role in shaping the accessibility and quality of cancer care.

To further elucidate the dynamics of chemotherapy access disparities, future research should include more diverse geographic settings and healthcare systems to enhance the generalizability of findings. They can examine the specific impact of socioeconomic and cultural factors on treatment access and adherence. They can explore the role of policy interventions in mitigating disparities, including insurance coverage expansions, healthcare provider incentives, and infrastructure investments in rural areas. Additional research can implement longitudinal studies better to understand the long-term outcomes of disparities in chemotherapy delivery.

## Conclusions

This systematic review and meta-analysis have highlighted a potential disparity in the administration of chemotherapy for patients with unresectable NSCLC between rural and urban settings. Despite the limitations of significant heterogeneity and possible publication bias, the results suggest a marginally lower likelihood of chemotherapy use in rural areas (pooled OR of 0.91, 95% CI: 0.83-1.00). However, the confidence interval borders the null effect, indicating that these findings should be interpreted cautiously. The trend toward a rural-urban treatment gap underscores the need for policy interventions and healthcare system modifications to ensure that all patients with unresectable NSCLC have equitable access to standard care, regardless of geographic location. Future research should clarify these findings with more extensive, more homogenous studies and explore the underlying factors contributing to this discrepancy to inform targeted strategies for improvement.
